# Patient physical condition and functional sequelae following hospitalization with COVID-19: A cross-sectional observational study

**DOI:** 10.1097/MD.0000000000041948

**Published:** 2025-03-28

**Authors:** Daniel Ángel García, Inmaculada Calvo Muñoz, Ismael Martínez Nicolás, Bianca Salmeri

**Affiliations:** a Faculty of Physiotherapy, Occupational Therapy and Podiatry, UCAM Catholic University of Murcia, Murcia, Spain; b Fundación para la Formación e Investigación Sanitarias de la Región de Murcia, Instituto Murciano de Investigación Biosanitaria Pascual Parrilla, Murcia, Spain.

**Keywords:** COVID-19, observational study, rehabilitation, short physical performance battery

## Abstract

After hospitalization caused by COVID-19, a high prevalence of physical deterioration has been observed, hence the importance of having tests to evaluate the functional status of patients and to be able to perform a partition and subsequent referral to the physiotherapy service. This cross-sectional observational study describes the physical status according to the short physical performance battery (SPPB) of patients admitted to the hospital setting for COVID-19 and to identify variables potentially related to this outcome. Thirty-six patients admitted to the hospital setting for COVID-19 in the first wave living in the community. Patients were evaluated with the SPPB, strength test, the International Physical Activity Questionnaire, the 1-minute sit-to stand, spirometry, the Barthel index, the Hospital Anxiety and Depression Scale, and other patient-related data were collected. We performed bivariate and regression analyses. A linear regression was fitted, having SPPB as a dependent variable to ascertain the impact of intensive care unit (ICU) admission on physical performance. Five variables were related to SPPB. There was a significant relationship between admission to the ICU and having a heart disease (*P* = .015), the level of physical activity (*P* = .049), number of years smoking (*P* = .029) and days of hospitalization (*P* = .005). A total of 22.22% of analyzed patients suffered frailty. SPPB is related to altered respiratory pattern, quadriceps strength, 1-minute sit-to-stand and FEV1, Barthel score, days of hospitalization and FEV/FVC ratio. Lack of association between ICU stay, age or sex with SPPB results differs from the results of other studies.

## 1. Introduction

The COVID-19 pandemic led to unprecedented infection and death rates worldwide.^[1]^ Beyond its health impact, it has also caused significant economic, social, and emotional disruption.^[2]^ Most infected individuals experience mild to moderate respiratory illness and recover without specialized treatment. However, some develop severe symptoms requiring intensive care.^[3]^ Elderly individuals and those with preexisting conditions, such as cardiovascular disease, diabetes, chronic respiratory disease, or cancer, are at higher risk of severe illness.^[4]^

Rehabilitation has played a crucial yet inconsistent role in the care of both hospitalized and discharged patients.^[5]^ The lack of clear guidelines and emerging challenges have contributed to this variability.^[6]^ While evidence on effective interventions for post-COVID-19 sequelae remains limited,^[7]^ many patients still face incomplete recovery, highlighting the need for targeted rehabilitation strategies. The first step in addressing this issue is proper assessment. Therefore, this study aims to evaluate the physical status of patients hospitalized during the first wave of COVID-19 using the short physical performance battery (SPPB) and to identify key factors associated with functional outcomes. Additionally, we examine the relationship between Intensive Care Unit (ICU) admission and these variables to further understand its impact on recovery.

## 2. Methods

A descriptive cross-sectional observational study was conducted. The study population was defined as patients who had been admitted to hospital for COVID-19 from March to December 2020.

The largest reference hospital in the Region of Murcia was selected, and recruitment was carried out by telephone using the records of the primary care centers of the Servicio Murciano de Salud (SMS, Health Service of Murcia, Spain). The inclusion criteria were patients over 18 years of age, discharged from the Hospital Clínico Universitario Virgen de la Arrixaca (HCUVA), and identified with a COVID-19 diagnosis (ICPC-2 code). Patients admitted to hospital referred to the Region of Murcia from a neighboring province and those cases which were imported from outside the region were excluded. In addition, the study excluded patients whose hospital admission was consequential to an initial COVID-19 admission outside the region, patients re-admitted or in “non-stable” condition in the community setting, patients who died after hospital discharge, and patients who did not voluntarily agree to participate in this study.

All patients have given their informed consent for participation in the research study respecting the ethical principles of the 2013 Helsinki declaration and the ethical protocol set by the Ethics and Research Commission of the Hospital (protocol acceptance code 2021-2-1-HCUVA). Prior to participation, all patients provided written informed consent, ensuring they understood the study’s objectives, procedures, and confidentiality measures. Patient data were anonymized and securely stored in accordance with General Data Protection Regulation guidelines to maintain privacy and ethical integrity.

### 2.1. Location and setting

The data were collected by 5 evaluators who were not involved in the protocol development or project implementation and who completed 3 training sessions.

The assessment protocol comprised a face-to-face assessment of physical condition together with the collection of socio-demographic data, comorbidities, use of medication, use of healthcare services and patient-completed questionnaires.

Prior to the assessment appointment, patients were asked not to do intense exercise on that day and not to smoke in the previous half hour, and they were sent an online form by e-mail containing the Barthel questionnaire, the Hospital Anxiety and Depression Scale (HADS), and the following information: age, sex, admission to ICU and days in ICU, health conditions (comorbidities), name and number of medications administered, days of hospitalization, and days since hospital discharge. Patients were provided with a contact phone number in case they had any questions. When the patients did not know how to complete the online form or were unable to do so, this was completed the day of the face-to-face assessment appointment with the help of the evaluators.

During the in-person visit, the measurements were taken in this order: SPPB, quadriceps strength of the dominant leg, International Physical Activity Questionnaire (IPAQ-SF), height, weight, spirometry and the 1-minute sit-to-stand test (1-MSTST). Patients were informed of the purpose of each test and were encouraged to ask the evaluator any questions they might have when needed.

The measuring materials used in the tests were: Lafayette Manual Muscle Testing System, model 01165, Instrument Company, US.^[8]^ Sibelmed DATOSPIR Touch Spirometer.

Once all the measurements had been taken, the participants were asked, as an open question, about possible sequelae resulting from the COVID-19 infection. Data collection was carried out from October 2021 to June 2022.

### 2.2. Variables

The main variable taken was SPPB, which comprises 3 tests: balance; gait speed; and getting up and sitting in a chair 5 times.^[9]^

The secondary variables considered were: spirometry results for forced vital capacity and forced expiratory volume in 1 second (FEV1)^[10,11]^; quadriceps strength according to Kendall, measured with dynamometer^[12]^; Barthel index^[13]^; habitual physical activity level measured through the IPAQ-SF^[14]^; 1-minute sit-to stand test (1-STST).^[15]^

The control variables studied were: age; sex; body mass index; ICU admission; previous health conditions worsening the course of the disease coded as number of comorbidities^[16]^; HADS score^[17]^; number of daily medications administered; days since hospital discharge at the time of study assessment; days of hospitalization. To select the variables, we based our choices on the proposal by Ejaz et al^[16]^ and consulted with an expert in respiratory physiotherapy and another expert in sports physiotherapy to assess whether additional variables needed to be included. These 2 experts were external to the study. The study authors ultimately validated the selected variables.

### 2.3. Bias

To avoid the influence of confounding variables, an attempt was made to collect all the variables identified in the literature that are related to COVID.

To address response bias and maximize the number of measurements possible, patients were given the option of being measured at home or at the UCAM facilities. In addition, a contact phone number was provided to assist in filling in the form and there was the option of completing the form in person.

The self-reported information bias was addressed by contacting patients when there was incorrect or missing information.

### 2.4. Sample size

As of 1 July 2020 (first wave), there were 709 admissions in the Region of Murcia, with 113 patients admitted to the ICU. The sample size calculated to detect 1-point changes in the SPPB score – taking the reference values for women published by Cabrero-García et al^[18]^; for 95% power, 5% alpha and adjusted for finite population – was 68 patients. The sample was taken from all subjects admitted with a diagnosis of COVID-19 by systematic random sampling. When there was no response after telephone contact or refusal to participate, the study subject was replaced by another subject using the same sampling mechanism.

### 2.5. Statistical analysis

The software used in the analysis was IBM SPSS Statistics 23.0. A descriptive analysis of the characteristics of the study participants was conducted. The values of quantitative variables were expressed as mean, standard deviation, median and range. For qualitative variables, values were summarized as absolute and relative frequencies.

### 2.6. Statistical methods

Data relating to subject-specific information and to the study variables were entered into a database created for this purpose (Microsoft Excel© 365). The data were recorded by 3 independent evaluators via forms. Two independent researchers reviewed, validated and provided feedback for error and missing cases detection to the evaluators. Unknown or improbable values were searched for by means of data cleaning with logic and range tests.

### 2.7. Analysis of the main and secondary variables

A bivariate analysis of correlation between quantitative variables was performed using Spearman test. Bonferroni adjustment was not used as it is not recommended for exploratory analysis.^[19]^

Subsequently, ICU admissions and non-ICU admissions were compared by means of independent group mean difference (Student *t* test) when the variable had a normal distribution, and Mann–Whitney *U*-test otherwise. The data were tested for normal distribution using the Shapiro–Wilk test. And the Chi-square test was used for qualitative variables. A significance level of 95% was taken for all statistical tests.

Finally, an exploratory regression analysis was performed to identify those variables related to the SPPB score. The first step was to perform a linear regression using the numerical score of the SPPB scale as the dependent variable. In the first instance, all variables of interest were included. The covariates were then selected in a stepwise process in order to obtain a more parsimonious model without overfitting, exploring the model to ensure the absence of multicollinearity,^[20]^ heteroskedasticity^[21]^ and misspecification.^[22]^ Only those variables that violated any of the regression assumptions were discarded.

## 3. Results

**Figure 1.**
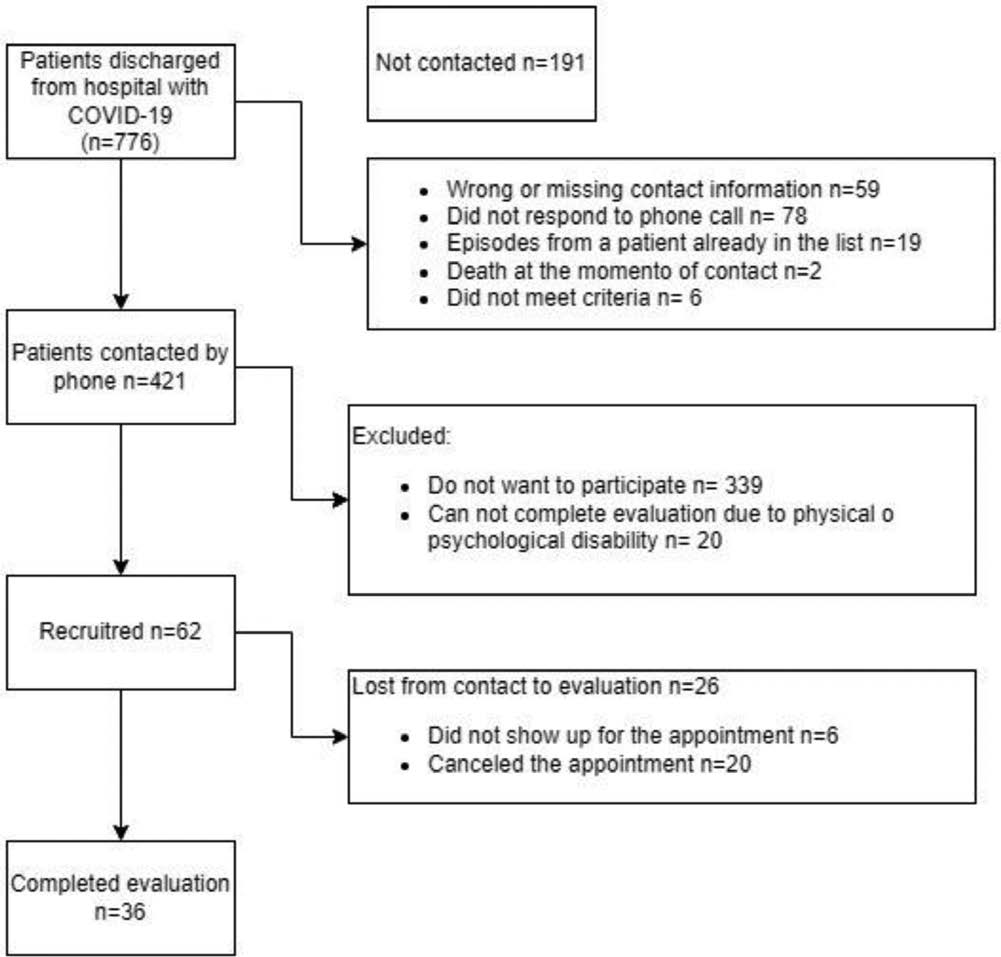
Patient identification and recruitment flow chart.

A total of 776 patients discharged from the HCUVA with a COVID-19 diagnosis were identified, out of whom 195 were not contacted and 164 did not answer the call for reasons shown in Figure 1. Among these, there were 6 minors – whose age was not recorded in the database – who did not meet the criteria. From those who were contacted, 359 did not want to participate or were unable to participate due to their physical or mental condition. Among the 62 who agreed to participate in the program in the first instance, 26 did not actually attend the measuring appointment. Finally, 36 patients were evaluated.

### 3.1. Descriptive

The characteristics of the 36 study subjects are reported in Tables 1 and 2. In summary, the sample had a mean age of 58.27 years (SD 13.53) and was predominantly male (55.56%). Only 28.57% reported no comorbidities – hypertension and respiratory pathologies were the ones most frequently reported. Ex-smokers (42.86%) had smoked for an average of 5.86 years (SD 12.22). The average number of days of hospitalization for COVID was 10.67 (2–45) and 19.04% (n = 5) of patients required admission to intensive care. 22.22% were found to be frail, 5.56% showed moderate dependency and 22.22% showed low dependency. In addition, approximately 1 third of the sample reported some degree of depression or anxiety.

**Table 1:** Descriptive data of the sample. continuous variables.

Variable	Median	Range	Mean	Standard deviation
Age (yr)	58.52	57.19 (27.53–84.72)	58.27	13.53
Size (cm)	163	42 (144–186)	163.79	10.36
Weight (kg)	82.5	65 (54–119)	82.78	18.02
BMI	29.82	25.54 (19.73–45.27)	29.70	6.93
Years of smoking	0	50 (0–50)	5.86	12.22
Years as an ex-smoker*	0	49 (0–49)	8.97	14.70
Days of hospitalization	8.00	43 (2–45)	10.67	8.89
Days in ICU†	13	3 to 30	2.19	6.52
Days since discharge	553	313 to 960	504.86	143.14
SPPB index	10.5	8 (4–12)	10.11	1.96
Barthel index	100	10 (90–100)	98.33	2.92
Quadriceps strength	240.68	603.70 (65.33–669.03)	267.24	134.90
FVC	3.12	6.42 (0.59–7.01)	3.29	1.17
FEV1	2.48	4.3 (0.50–4.8)	2.53	0.90
FEV1/FVC	0.81	0.47 (0.44–0.91)	0.78	0.10
HADS – anxiety	10.5	14 (4–18)	10.75	3.89
HADS – depression	8.5	9 (5–14)	8.50	1.99
No. of comorbidities	1	5 (0–5)	1.27	1.32
No of medications administered	2.5	13 (0–13)	3.77	3.69
1-MSTST	25	57 (1–58)	25.83	10.65
Total METS	1566	17,731 (80–17,811)	2589.78	3392.89

1-MSTST = 1-minute sit-to stand test, BMI = body mass index, FEV1 = forced expiratory volume in the 1st second, FVC = forced vital capacity, HADS = hospital anxiety and depression scale, ICU = Intensive Care Unit, METS = metabolic equivalent of task, N = Newton, SPPB = short physical performance battery.

* Data on ex-smokers.

† Data on patients admitted to the ICU.

**Table 2:** Sample description: categorical variables.

Variable	Frequency	%
*Sex*		
Female	16	44.44
Male	20	55.56
*BMI*		
Underweight	1	2.78
Normal weight	2	8.33
Overweight	16	44.44
Obesity	17	47.22
*Smoking*		
Ex-smoker	14	38.89
Nonsmoker	22	61.11
*Comorbidities*		
Asthma	3	8.33
Autoimmune diseases	2	5.56
Cancer	4	11.11
Cardiovascular diseases	10	27.78
Diabetes	4	11.11
Hypertension	12	33.33
Respiratory diseases	11	30.56
None	11	30.56
*Dominant leg*		
Right	24	66.67
Left	12	33.33
*SPPB*		
Balance		
Held in semi-tandem 10s and in tandem 3 to 9 s	4	11.11
Held in tandem 10 s	32	88.89
Gait speed		
>8.7 s	1	2.78
Between 8.7 and 6.21 s	7	19.44
Between 6.2 and 4.82 s	8	22.22
<4.82 s	20	55.56
Five-times sit-to stand		
Cannot	1	2.78
Greater than or equal to 16.7 s	6	16.67
Between 16.6 and 13.7 s	4	11.11
Between 13.6 and 11.2 s	9	25.00
Less than or equal to 11.1 s	16	44.44
*Frailty (SPPB < 10*)		
Not frail	28	77.78
Frail (<10)	8	22.22
*Dependency degree* *		
Moderate	2	5.56
Low	8	22.22
Independent	26	72.22
*Physical activity level* †		
Low	8	22.22
Moderate	21	58.33
High	7	19.44
*Breathing pattern* ‡		
Regular	14	38.89
Obstructive	5	13.89
Resistive	6	16.67
Mixed	9	25.00
*Breathing pattern* §		
Regular	14	38.89
Obstructive	7	19.44
Resistive	14	38.89
Mixed	1	2.78
*Anxiety level* ∥		
Healthy	9	25.00
Little	9	25.00
Moderate	11	30.56
Serious	7	19.44
*Depression level* ∥		
Healthy	11	30.56
Little	18	50.00
Moderate	7	19.44

BMI = body mass index, IPAQ-SF = international physical activity questionnaire-short form, SPPB = short physical performance battery.

* According to Barthel index.

† According to IPAQ-SF questionnaire.

‡ According to Romero de Avila Cabezona et al.

§ According to Miller quadrants.

∥ According to hospital depression and anxiety scale.

Regarding comorbidities 12 patients had hypertension, 11 had respiratory diseases, 10 had cardiovascular pathologies, 4 had a history of cancer, 4 had diabetes, 3 had asthma and 2 had autoimmune diseases. There were 11 patients with no comorbidity.

### 3.2. Main results

#### 3.2.1. Bivariate relationships

Variables related to SPPB in a bivariate manner are altered breathing pattern (<80% of the theoretical value) of FEV1 (*P* = .026), days since discharge (*P* = .002), quadriceps strength (*P* = .010), 1-MSTST score (*P* = .011), FEV1 (*P* = .011). In addition, there was a significant relationship between admission to the ICU and having a heart disease (*P* = .015), the level of physical activity (*P* = .049), number of years smoking (*P* = .029) and days of hospitalization (*P* = .005).

### 3.3. Regression

The regression variables were selected to represent the usual way of using them in the clinic, without scale transformations. For instance, the values of scales such as Barthel were taken as continuous numerical values (values from 1 to 100) and the level of physical activity was used as low, moderate and high categories.

Once the variables that did not meet the regression assumptions mentioned in the methodology were removed, the regression shown in Table 3 was obtained. Only the Barthel score (0.266, *P* = .037), the days of hospitalization (−0.077, *P* = .043) and the FEV/FVC ratio (7.299, *P* = .017) showed a significant relationship (*P* < .05) with the SPPB.

**Table 3:** Multivariate regression.

SPPB index	Coef.	Std. Err.	*t*	*P*> *t*	[95% Conf. Interval] inf	[95% Conf. Interval] sup
Sex	−0.628	0.624	−1.010	.325	−1.923	0.666
Age	−0.025	0.024	−1.050	.303	−0.074	0.024
BMI	−0.061	0.048	−1.270	.219	−0.160	0.039
Years of smoking	0.045	0.024	1.860	.077	−0.005	0.096
No. of comorbidities	0.257	0.320	0.800	.431	−0.407	0.920
No. of medications administered	−0.118	0.101	−1.170	.254	−0.328	0.091
Days since discharge	0.000	0.002	−0.070	.947	−0.004	0.004
Days of hospitalization	−0.077	0.036	−2.140	**.043**	−0.152	−0.003
HADS – anxiety	0.025	0.150	0.160	.871	−0.286	0.335
HADS – depression	0.015	0.089	0.170	.864	−0.169	0.200
Total METS	0.000	0.000	−0.230	.817	0.000	0.000
Barthel	0.266	0.120	2.220	**.037**	0.017	0.516
FEV1/FVC	7.299	2.835	2.570	**.017**	1.418	13.179
Cons.	−17.770	12.544	−1.420	.171	−43.784	8.244

Bold values indicate *P* < .05.

BMI = body mass index, coef. = coefficient, cons = constant, FEV1 = forced expiratory volume in the 1st second, FVC = forced vital capacity, HADS = hospital anxiety and depression scale, METS = metabolic equivalent of task, no. = number, SPPB = short physical performance battery, Std. Err. = standard error.

## 4. Discussion

The SPPB results revealed 22.22% of people in the sample assessed were in the process of developing frailty. On a bivariate basis the result on the SPPB is related to physical variables such as quadriceps strength, 1MSTS and spirometry values, as well as time from discharge to assessment. When the multivariate analysis is performed eliminating variables that do not comply with the principle of non-collinearity, the exploratory model leaves Barthel score, days of hospitalization and FEV/FVC ratio as variables significantly related by SPPB.

In the multivariate model, smoking history shows a tendency to influence the SPPB score as well. Interestingly, age is not a variable related to the SPPB score in any of the 2 analyses, as this is contrary to the results of other studies.^[23]^ Several factors may explain this discrepancy. First, the characteristics of our sample could have influenced the results. Our cohort had a mean age of 58.27 years, which is younger than in other studies where frailty and functional decline are more pronounced in older populations. The inclusion of younger individuals may have reduced the impact of age as a determining factor for SPPB scores. Second, methodological differences could contribute to this divergence. While some studies assess functional decline using broader frailty indices or different mobility assessments, our study focused specifically on SPPB, which, although widely validated, may not capture subtle functional impairments in all patient subgroups. These results should make us reflect on whether we should only assess patients over 65 years of age as those who may potentially have a loss of muscle and functional capacities during their stay in hospital, as recommended,^[24]^ or extend this assessment to everyone admitted or at least with multiple risk factors.

These findings align with other observed associations in our study, particularly regarding ICU admission and its relationship with key patient characteristics. Although our design does not allow for causal inference, existing literature provides insight into possible mechanisms. Higher levels of pre-COVID-19 physical activity may enhance physiological resilience, reducing disease severity and the likelihood of ICU admission.^[25]^ Conversely, preexisting cardiovascular disease exacerbates COVID-19 outcomes through systemic inflammation, endothelial dysfunction, and prothrombotic states, increasing the risk of severe complications.^[26]^ Additionally, smoking history is associated with impaired lung function and chronic inflammation, which may contribute to worse pulmonary outcomes and greater ICU admission rates.^[27]^ While these findings are consistent with prior research, further studies with larger sample sizes are needed to better establish the strength and direction of these associations. Furthermore, post-discharge recovery factors may have played a role in minimizing differences between ICU and non-ICU patients at the time of assessment. While ICU admission is often linked to worse functional outcomes, targeted rehabilitation efforts and prolonged recovery time may mitigate these effects, particularly in patients with better pre-hospitalization health status. These factors suggest that while ICU admission is a marker of acute severity, its long-term functional impact may vary depending on individual recovery trajectories.

The cutoff point of the SPPB scale for the onset of frailty^[28]^ coincides with other cutoffs, e.g., the 1 identifying functional limitations.^[29]^ This reference is important to clarify the working guidelines of the rehabilitation professionals. Establishing assessments to determine the care needs of patients in relation to their physical condition and functionality is a role that rehabilitation must urgently develop in the hospital setting. Evidence suggests that a patient bedridden in hospital can lose up to 40% of their muscle mass, which clearly needs to be seen as a patient safety issue.^[30,31]^ When admitting a patient will lead to a decline in their functional status down to levels that are dangerous to their health and independence, it is up to the system to address that situation. The presence of frailty in our sample is clinically relevant, as it has been linked to higher risks of falls, loss of independence, and increased healthcare utilization. These findings emphasize the importance of early screening and rehabilitation strategies and the implementation of measures focusing on strength, balance, and mobility training, while integrating physiotherapy with nutritional and lifestyle interventions to optimize recovery and long-term outcomes. Key moments would be at admission, to identify patients at high risk of falls or early frailty^[32]^; at scheduled stays of 1 week or more, to prevent loss of muscle mass and associated functional deficit^[33]^; and at discharge, to identify whether the patient needs follow-up at primary care or home care because they are dependent or at risk of frailty, thus avoiding future hospitalizations.^[34]^

The results obtained support the hypothesis that COVID-19 sequelae may persist, possibly suggesting that the disease weighs on different aspects of the functional independence of patients. Our results are similar in incidence of frailty to those of other studies.^[35,36]^ The healthcare system cannot afford the expense, either personal or financial, of an admitted patient acquiring any degree of frailty due to their hospital stay.

Our findings highlight the relevance of SPPB as a functional assessment tool, and the possibility to use associated measures like quadriceps strength, FEV1 or 1-MSTST. The bivariate and regression associations suggest that rehabilitation strategies should prioritize strength training, respiratory therapy, and functional mobility exercises to mitigate long-term sequelae in post-hospitalized COVID-19 patients.

In addition to rehabilitation, these findings reinforce the need for a preventive approach within healthcare systems, addressing risk factors before hospitalization occurs. Given the protective role of habitual physical activity, public health policies should emphasize the promotion of regular exercise as a primary strategy to enhance resilience against severe infections like COVID-19. Likewise, the negative impact of smoking on pulmonary function and overall health highlights the importance of stronger smoking cessation programs. Preventive strategies, such as community-based physical activity initiatives and targeted smoking reduction campaigns, could contribute to reducing hospital admissions and improving long-term health outcomes, ultimately lessening the burden on healthcare systems.

One limitation of our study was the low response rate of patients. Only 6% of the contacted patients actually attended the assessment. Even though this meant measuring less sample than planned, the lesson to be learned from this is that patient displacement appears to be difficult, reinforcing the idea of identifying patients at risk in the same hospital or working in collaboration with primary care to reduce follow-up loss. Moreover, this way we would be contributing to reducing the environmental impact of healthcare, an aspect that will increasingly govern our actions in a more direct way.^[37]^ Although our sample size of 36 patients is relatively small, the fact that significant associations emerged despite this limitation suggests that the observed effects are robust. The significant associations observed – despite the limited number of participants – suggest that the effects are robust and merit deeper exploration in larger samples. By focusing on these primary determinants, our study provides a solid foundation for future research to further explore subtle associations in larger cohorts.

All these ideas confirm the need to shift the rehabilitation model towards a proactive recruitment of patients, offering rehabilitation to those who need it in order to avoid future costs and preventable morbidity.^[38]^

Our study provides a cross-sectional snapshot of post-COVID-19 functional sequelae, but the long-term trajectory of recovery was not explored. Future studies could deepen in potential confounders that might be missed, incorporating longitudinal follow-ups at multiple time points to track recovery patterns, assess the effectiveness of rehabilitation strategies, and identify patients who may require prolonged intervention. Our research group aims to establish routine assessments for at-risk patients within our healthcare system. We have already initiated collaborations with hospital physiotherapy teams and regional health authorities to implement these measures.

In conclusion according to the SPPB cut points, COVID hospitalization had an impact on the physical condition of some patients, with 22.22% of people showing a process of frailty.

SPPB is related to the altered respiratory pattern of FEV1, days since discharge, quadriceps strength, 1-MSTST score and FEV1 in a bivariate analysis; and to the Barthel score, days of hospitalization and FEV/FVC ratio in multivariate test.

Patients admitted to the ICU showed a higher number of days of hospitalization, as well as a significant relationship with smoking, heart disease and level of habitual physical activity. No increased symptom severity was detected in patients admitted to the ICU.

## Author contributions

**Conceptualization:** Daniel Ángel García, Inmaculada Calvo Muñoz, Bianca Salmeri.

**Data curation:** Daniel Ángel García, Ismael Martínez Nicolás, Bianca Salmeri.

**Formal analysis:** Daniel Ángel García, Ismael Martínez Nicolás.

**Funding acquisition:** Daniel Ángel García.

**Investigation:** Daniel Ángel García, Bianca Salmeri.

**Methodology:** Daniel Ángel García, Inmaculada Calvo Muñoz, Ismael Martínez Nicolás.

**Project administration:** Daniel Ángel García, Inmaculada Calvo Muñoz.

**Resources:** Inmaculada Calvo Muñoz, Ismael Martínez Nicolás, Bianca Salmeri.

**Supervision:** Daniel Ángel García.

**Validation:** Daniel Ángel García, Ismael Martínez Nicolás.

**Writing** – **original draft:** Daniel Ángel García, Ismael Martínez Nicolás, Bianca Salmeri.

**Writing** – **review & editing:** Daniel Ángel García, Inmaculada Calvo Muñoz, Ismael Martínez Nicolás, Bianca Salmeri.
